# Analysis of abiotic and biotic stress-induced Ca^2+^ transients in the crop species *Solanum tuberosum*

**DOI:** 10.1038/s41598-024-79134-3

**Published:** 2024-11-11

**Authors:** Annelotte van Dieren, Roland E. Schwarzenbacher, Sophia Sonnewald, Andras Bittner, Ute C. Vothknecht

**Affiliations:** 1 Institute for Cellular and Molecular Botany, University of Bonn, Kirschallee 1, 53115 Bonn, Germany; 2 Department of Biosciences, Durham University, South Road, Durham, DH1 3LE UK; 3 Department of Biology, Chair of Biochemistry, Friedrich-Alexander-University Erlangen-Nuremberg, Staudtstraße 5, Erlangen, 91058 Germany

**Keywords:** Potato, *Arabidopsis thaliana*, Crops, Calcium signalling, Aequorin, Stress perception, Plant signalling, Plant sciences, Plant stress responses, Abiotic, Biotic

## Abstract

Secondary messengers, such as calcium ions (Ca^2+^), are integral parts of a system that transduces environmental stimuli into appropriate cellular responses. Different abiotic and biotic stresses as well as developmental processes trigger temporal increases in cytosolic free Ca^2+^ levels by an influx from external and internal stores. Stimulus-specificity is obtained by a certain amplitude, duration, oscillation and localisation of the response. Most knowledge on stress-specific Ca^2+^ transient, called calcium signatures, has been gained in the model plant *Arabidopsis thaliana*, while reports about stress-related Ca^2+^ signalling in crop plants are comparatively scarce. In this study, we introduced the Ca^2+^ biosensor apoaequorin into potato (*Solanum tuberosum*, Lcv. Désirée). We observed dose-dependent calcium signatures in response to a series of stress stimuli, including H_2_O_2_, NaCl, mannitol and pathogen-associated molecular patterns (PAMPs) with stimuli-specific kinetics. Direct comparison with Arabidopsis revealed differences in the kinetics and amplitude of Ca^2+^ transients between both species, implying species-specific sensitivity for different stress conditions. The potato line generated in this work provides a useful tool for further investigations on stress-induced signalling pathways, which could contribute to the generation of novel, stress-tolerant potato varieties.

**Supplementary Information:**

The online version contains supplementary material available at 10.1038/s41598-024-79134-3.

## Introduction

Plants sense and respond to unfavourable environmental conditions to mitigate the negative effects of such stresses. Indeed, each stress type and each stress combination cause a unique molecular footprint, which induces a fine-tuned cellular response^1,2^. For example, unique stress-specific transcriptional changes have been observed in response to heat, drought, high light, or combinations of these treatments in the model plant *A. thaliana*^3,4^. Secondary messengers are involved in transducing a primary stimulus into an appropriate cellular response. In plants, changes in the concentration of free Ca^2+^ in the cytosol (referred here as [Ca^2+^]_cyt_), but also in various organelles, have emerged as a universal secondary messenger^5–7^. A wide range of stresses as well as developmental processes trigger temporal increases in [Ca^2+^]_cyt_ by an influx of Ca^2+^ from external and internal stores. The amplitude and timing of Ca^2+^ influx depends on the type of perturbation, the magnitude of the stress and how often the plant faced such stress conditions in the past^8,9^, resulting in an information encoding ‘calcium signature’^10,11^. So far, calcium signatures have been described in Arabidopsis, for instance in response to oxidative stress, biotic elicitors, osmotic stress, salt, cold or touch^9,12–16^.

The use of the genetically encoded Ca^2+^ indicator (GECI) apoaequorin to determine absolute concentrations of free Ca^2+^ in plants was established in the 1990s in Arabidopsis^12^ and has since been used to investigate Ca^2+^ changes in many studies^17^. However, only few studies have reported the use of GECIs in crop species, such as H_2_O_2_ and NaCl responses in rice roots^18^, chilling response in winter wheat^19^, infection with *Cuscuta reflexa* in tomato^20^, and a set of stressors in barley^21^. The comparison of Ca^2+^ transients between those species revealed tissue- and species-specific calcium signatures in response to different stress stimuli, which might be related to species-specific sensitivity to each stressor or to differences in the down-stream responses.

Plants show different sensitivity to environmental factors depending on their genetic make-up, which defines stress adaptation and acclimation. Potatoes are cultivated in nearly all regions of the world and are an important factor for global nutrition^22^. However, potato plants originate from an area with particular conditions, the high altitudes of the Andes. Based on its origin, potato growth, and by that also tuber yield, is quite sensitive to a wide range of environmental factors, including elevated temperatures^23^ and salinity^24^. So far, Ca^2+^ signals have only been investigated in response to flagellin 28 (flg-28) in potato plants^25^. To obtain a more comprehensive overview, we generated transgenic potato lines expressing apoaequorin under the control of the cauliflower mosaic virus (CaMV) 35S promoter and determined calcium signatures in response to different abiotic and biotic stimuli in leaf tissue of soil-grown plants. We performed direct comparisons with Ca^2+^ responses in Arabidopsis in order to put the results obtained for potato in context of the vast knowledge available from this model plant.

## Results

### Generation and characterisation of transgenic potato lines expressing *apoaequorin*

**Fig. 1:**
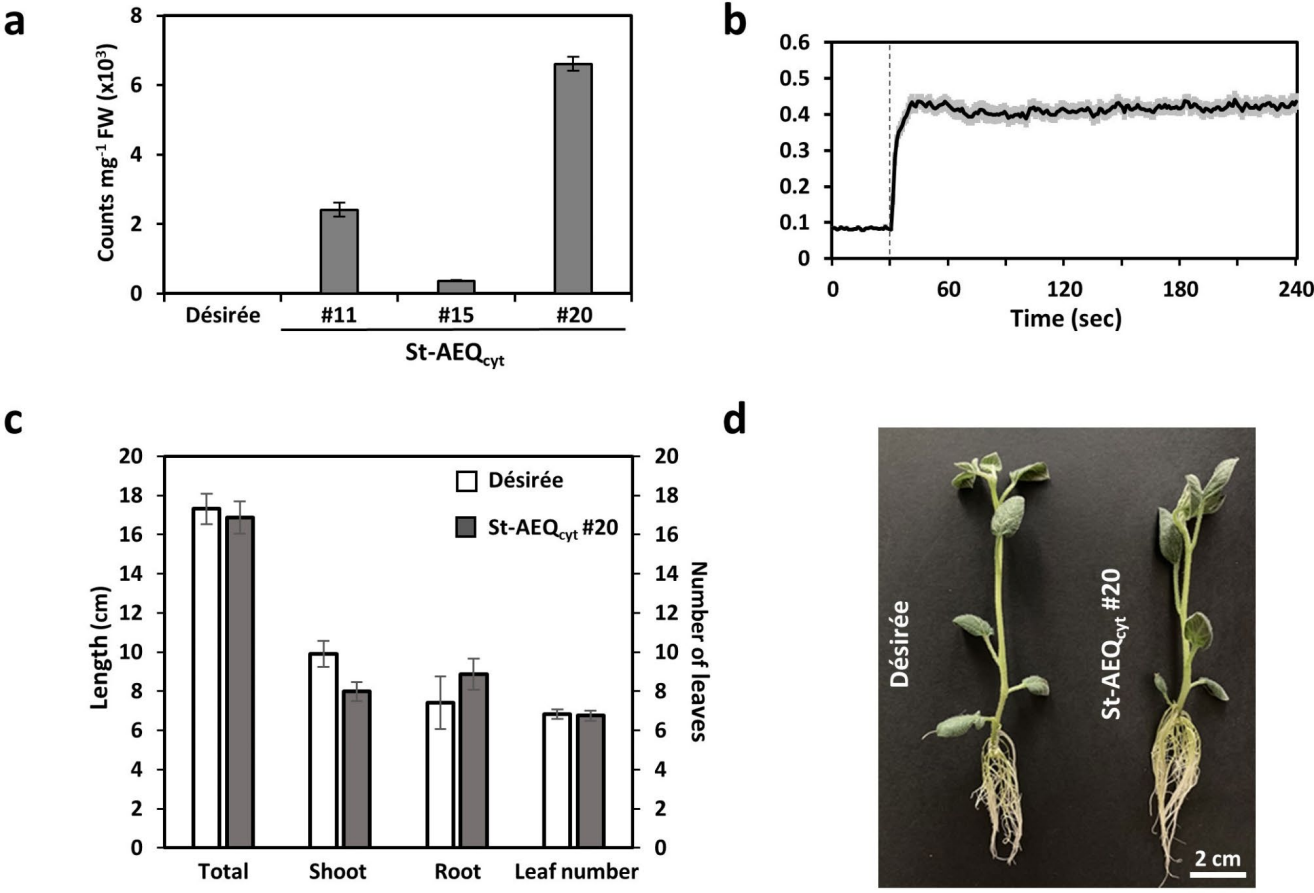
Selection and characterisation of St-AEQ_cyt_ lines. (**a**) Photon counts (average counts 10 s after adding 50 mM CaCl_2_) were measured in leaf extracts from three independent transgenic potato St-AEQ_cyt_ lines after in vitro reconstitution of aequorin with coelenterazine. Untransformed potato cultivar Désirée was used as a negative control (*n* = 9, mean ± SE). (**b**) Time course of changes in [Ca^2+^]_cyt_ in leaf discs from St-AEQcyt #20 in response to external Ca^2+^ application (final concentration 500 mM). (**c**) Comparison of the total plant length, root length, shoot length, and number of leaves in three-week old St-AEQ_cyt_ #20 and wildtype Désirée plants (*n* = 8, mean ± SE). (**d**) Representative picture of three-week old Désirée wild type and St-AEQ_cyt_ #20 plant.

To measure stimuli-induced [Ca^2+^]_cyt_ changes in vivo in potato plants, three independent transgenic potato lines (*Solanum tuberosum* L. cv. Désirée) carrying the p35S::apoaequorin construct were created (Fig. 1; Supplementary Fig. S1), referred to hereafter as St-AEQ_cyt_. Protein extracts from all lines showed aequorin luminescence in response to discharge solution (25 mM CaCl_2_) after overnight reconstitution with coelenterazine (Fig. 1a) indicating expression of the apoaequorin protein. Different luminescence levels were observed among the three St-AEQ_cyt_ lines, with #20 showing the highest luminescence level, and this line was therefore used for all further experiments. Functionality of the sensor *in planta* could be confirmed by measuring Ca^2+^ induced Ca^2+^ transients (Fig. 1b). Importantly, the insertion of the construct into the genome and expression of apoaequorin did not have any visible impact on growth and development in comparison to wild type plants (Fig. 1c and d). Western Blot analysis confirmed the presence of the apoaequorin protein in leaf extract (Supplementary Fig. S1a). The comparison to a previously described Arabidopsis line carrying the same *35S::apoaequorin* construct (At-AEQ_cyt_) showed a lower expression level of apoaequorin (Supplementary Fig. S1a). Accordingly, only about 25% of the aequorin luminescence in response to discharge solution was observed in St-AEQ_cyt_ lines compared to At-AEQ_cyt_ (Supplementary Fig. S1b).

### Calcium signatures in Arabidopsis and potato in response to abiotic stimuli

**Fig. 2:**
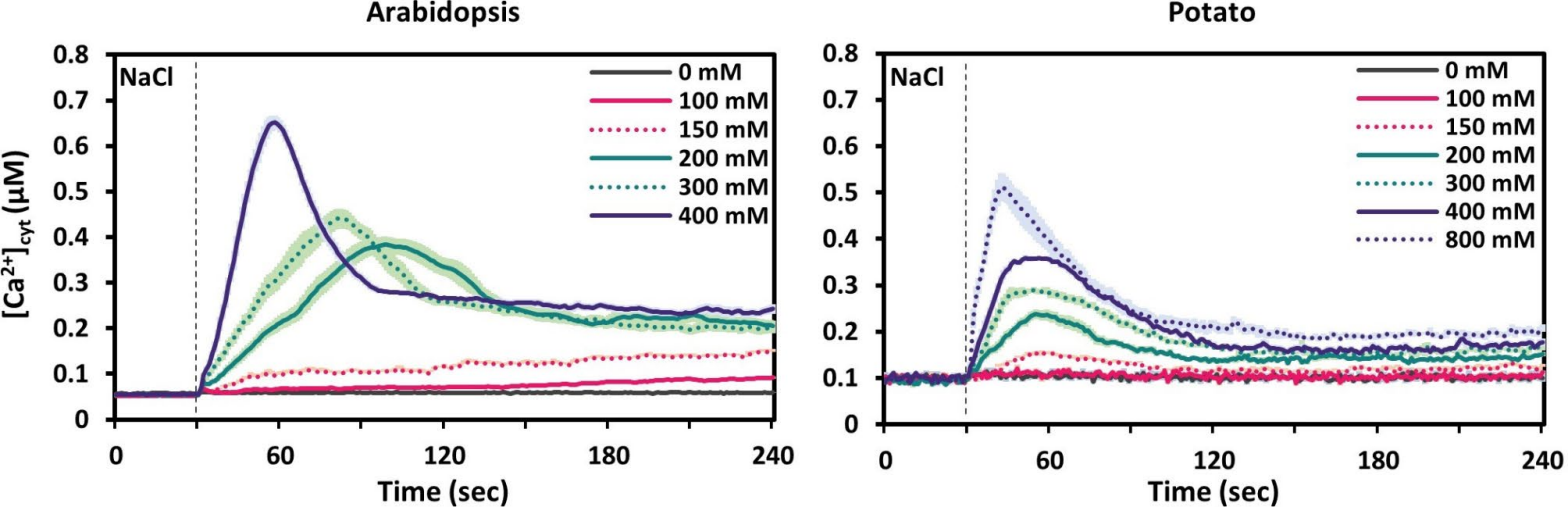
Time course of changes in [Ca^2+^]_cyt_ in response to different concentrations of NaCl in leaf tissue of Arabidopsis (left) and potato (right) plants. Values are shown as mean ± SE (*n* = 9). Dashed vertical lines indicate the time point of stimuli application (30 s).

**Fig. 3:**
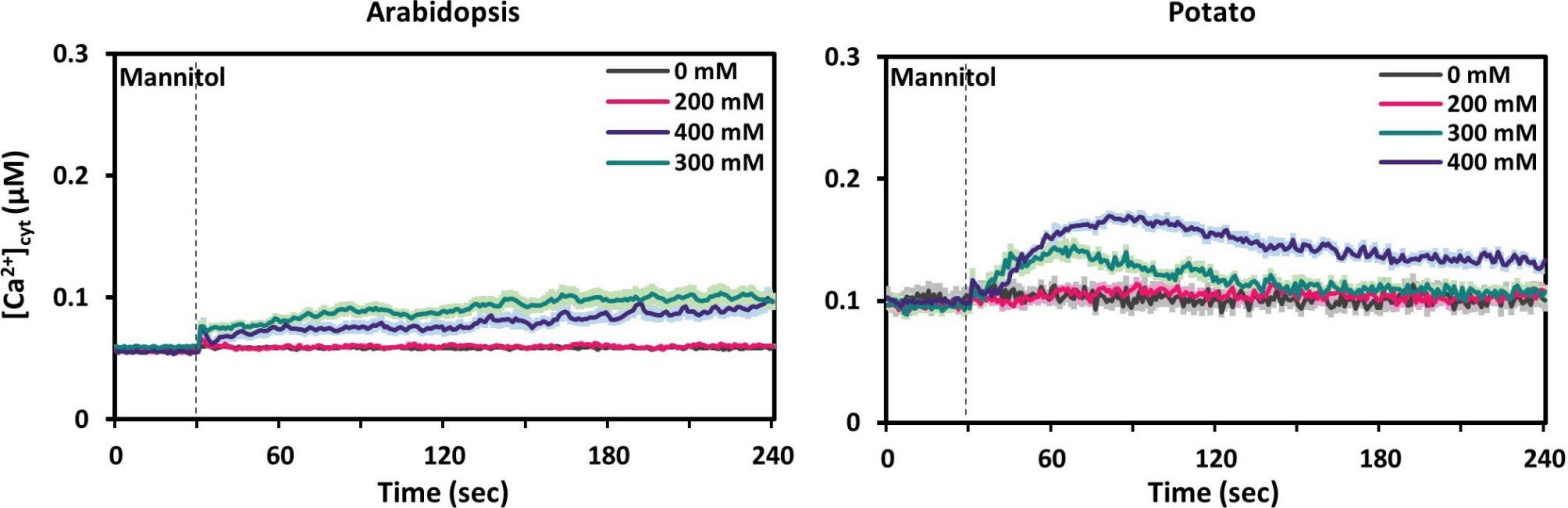
Time course of changes in [Ca^2+^]_cyt_ in response to different concentrations of mannitol in leaf tissue of Arabidopsis (left) and potato (right) plants. Values are shown as mean ± SE (*n* = 9). Dashed vertical lines indicate the time point of stimuli application (30 s).

**Fig. 4:**
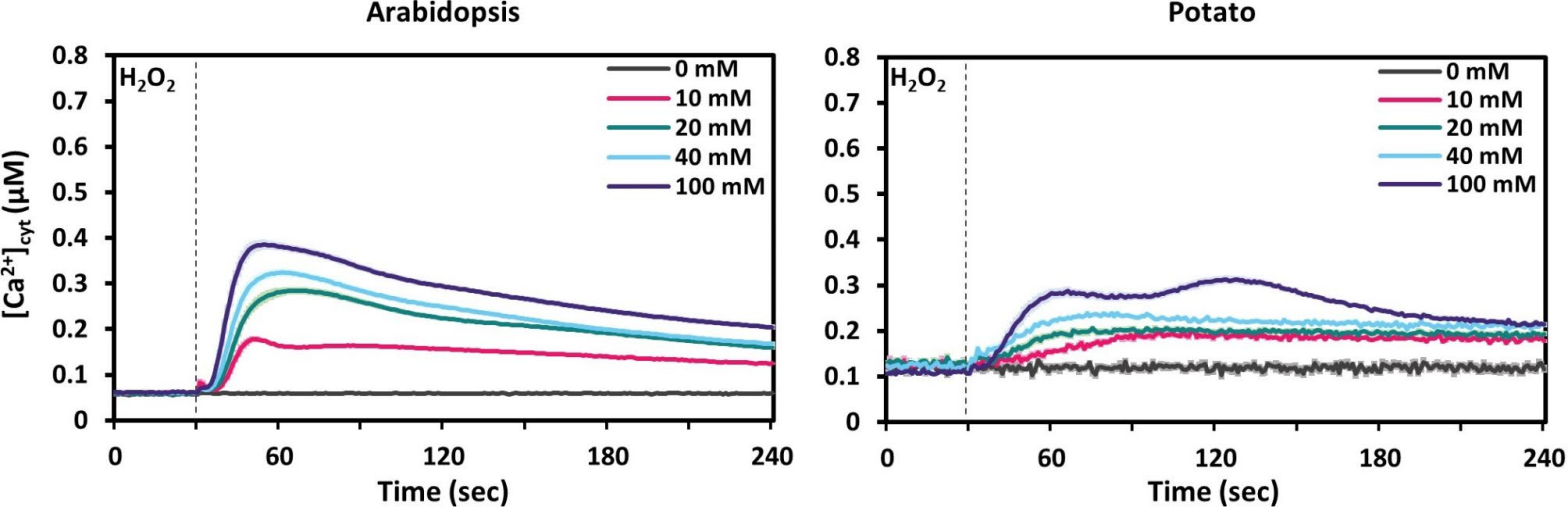
Time course of changes in [Ca^2+^]_cyt_ in response to different concentrations of H_2_O_2_ in leaf tissue of Arabidopsis (left) and potato (right) plants. Values are shown as mean ± SE (*n* = 9). Dashed vertical lines indicate the time point of stimuli application (30 s).

We first analysed stress-induced calcium signatures in leaf discs of Arabidopsis (At-AEQ_cyt_) and potato (St-AEQ_cyt_) in response to the abiotic factors salinity, drought and oxidative stress (Figs. 2, 3 and 4). To that end we treated leaf discs from plants of similar age grown on soil with NaCl, mannitol and H_2_O_2_.

### Salt stress (NaCl)

Initially the salt response was tested in steps of a two-fold increase starting with 100 mM NaCl (Fig. 2). In both species, NaCl elicited a strong response after application of concentrations above 200 mM NaCl, but the kinetics of the response differed between the two species. In Arabidopsis, 200 mM NaCl produced a broad peak occurring at around 70 s after application with a maximum [Ca^2+^]_cyt_ amplitude of 0.38 µM (Fig. 2, Arabidopsis, green solid line ). Increasing the NaCl concentration to 400 mM further enhanced the amplitude to 0.65 µM [Ca^2+^]_cyt_ and shifted the time of the maximum peak to around 30 s after application (Fig. 2, Arabidopsis, purple solid line). The shift in the timepoint of the peak value in response to increasing NaCl concentrations made us decide to test the response also to intermediate NaCl concentrations (Fig. 2, Arabidopsis, dotted lines), resulting in true interjacent responses. By testing all these different concentrations, we were able to visualise the dose-dependent shaping of the curve towards a stronger and faster response. Neither time nor amplitude of the maximum peak value did alter any more at concentrations above 400 mM.

In potato, application of NaCl also triggered clear Ca^2+^ transients (Fig. 2, potato), albeit with a slightly lower amplitude than in Arabidopsis. Also potato showed a dose-dependent increase in the amplitude of the [Ca^2+^]_cyt_ peak, however, the timing of the [Ca^2+^]_cyt_ peak only showed a very minor shift (Fig. 2, potato). In contrast to Arabidopsis, 800 mM NaCl (Fig. 2, potato, purple dotted line) was needed to induce a maximum response of 0.5 µM [Ca^2+^]_cyt_, comparable in height and shape to the response to 400 mM NaCl in Arabidopsis. Overall, these data show that dose-dependent NaCl-induced Ca^2+^ transients occur in both species, but they show differences in their kinetics.

### Osmotic stress (Mannitol)

To investigate the calcium signature of Arabidopsis and potato in response to acute osmotic changes, we recorded free [Ca^2+^]_cyt_ in leaf discs upon application of different concentrations of mannitol (Fig. 3). Because of the crystallizing properties of mannitol at higher concentrations, and the fact that we have to inject a two-fold concentrated stock solution via a narrow syringe system, the concentrations that we could test were limited to a maximum of 400 mM.

In Arabidopsis, a mannitol application with a concentration of 200 mM did not result in any response. Also higher concentrations (300 and 400 mM) did not induced a clear Ca^2+^ spike but led to a long-lasting minor elevation of [Ca^2+^]_cyt_ throughout the whole measurement period (240 s). This elevation started immediately after application of the stimulus and steadily increased to about 0.05 µM [Ca^2+^]_cyt_ (Fig. 3, Arabidopsis). In contrast to Arabidopsis, we observed a small but clear Ca^2+^ spike in potato at a concentration of 300 mM with a maximum amplitude of 0.15 µM [Ca^2+^]_cyt_ at about 30 s after application (Fig. 3, potato). At 400 mM the amplitude of the response increased further, and the peak value was reached later (60 s after application).

### Oxidative stress (H_2_O_2_)

Oxidative stress is a common result of unfavourable growth conditions but also certain biotic stimuli induce an oxidative burst^26^. Hydrogen peroxide (H_2_O_2_) is considered as the predominant signalling molecule during oxidative stress, due to its stable nature (half-life > 1 ms) compared to other reactive oxygen species^27^. Therefore, we used H_2_O_2_ as a stimulus to analyse [Ca^2+^]_cyt_ changes in response to oxidative stress (Fig. 4).

In Arabidopsis, H_2_O_2_ treatment with the lowest tested concentration (10 mM) resulted directly in a clear peak-shaped response with a maximum amplitude of 0.2 µM [Ca^2+^]_cyt_ just 15 s after application. The peak value of [Ca^2+^]_cyt_ increased in a dose-dependent manner with a maximal response of 0.4 µM observed at 100 mM (Fig. 4, Arabidopsis, purple line). In potato, 10 mM H_2_O_2_ did not elicit a clear Ca^2+^ transient but a slow minor increase (Fig. 4, potato, pink line) similar to what was observed in Arabidopsis in case of mannitol. At 20 mM H_2_O_2_ a peak-like shape started to appear, which turned into a double-peaked calcium signature at 100 mM H_2_O_2_ (Fig. 4, potato, purple line). The first peak occurred at around 30 s after application, similar to the peak seen in Arabidopsis, however, its amplitude was much lower (0.3 µM). The second peak was slightly higher than the first, occurred at around 90 s after application, was not visible in Arabidopsis plants (Fig. 4, Arabidopsis), and was also not seen in barley^21^.

### Calcium signatures in response to PAMPs

**Fig. 5:**
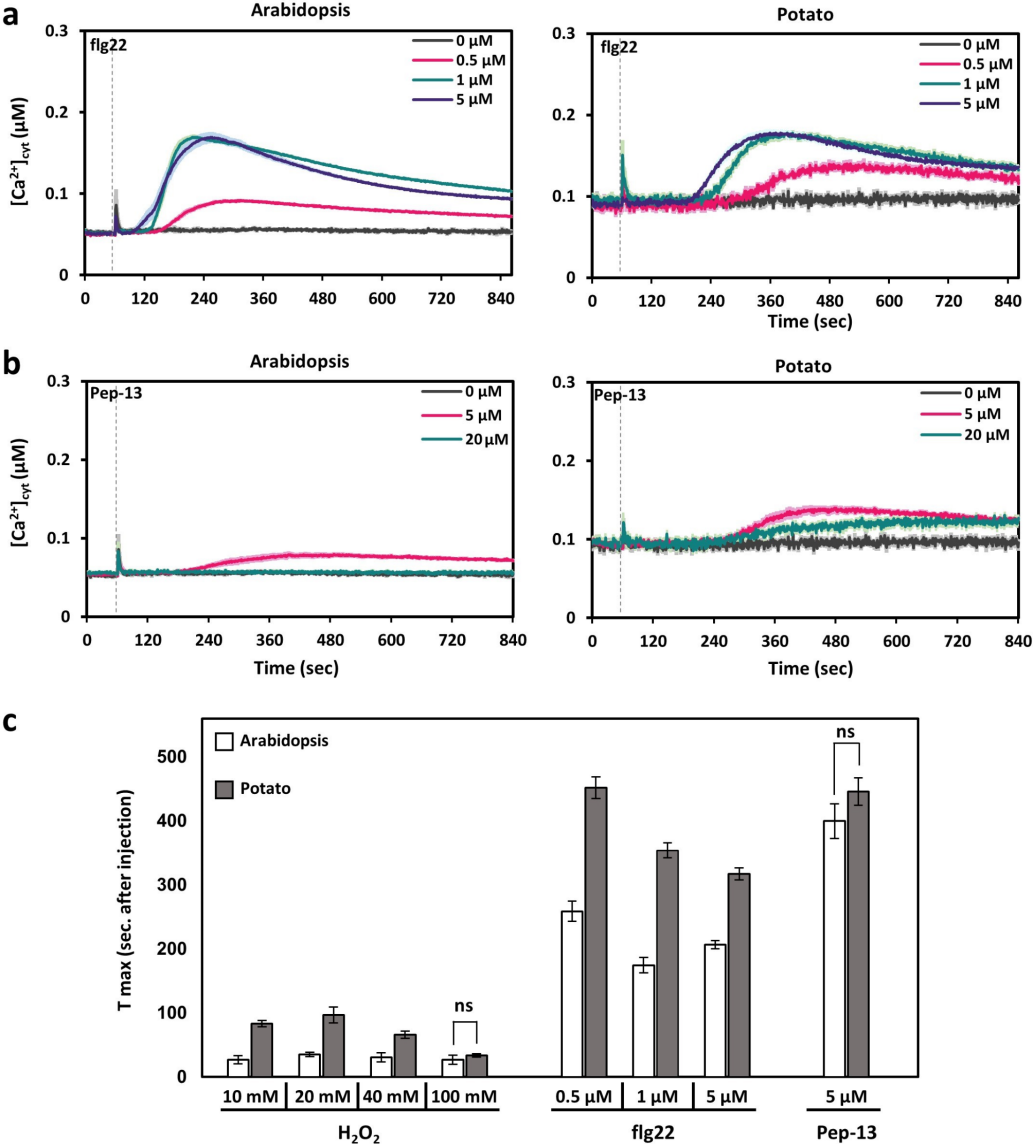
Induction of Ca^2+^ signals in response to pathogen-associated molecular patterns (PAMPs) in leaf tissue of Arabidopsis and potato. (**a**) Time course of [Ca^2+^]_cyt_ in Arabidopsis (left) and potato (right) in response do different concentrations of flg22. (**b**) Time course of [Ca^2+^]_cyt_ in Arabidopsis (left) and potato (right) in response do different concentrations of Pep-13. Values are shown as mean ± SE (*n* = 9). Dashed lines indicate the time point of PAMP application (60 s). (**c**) Time to reach the maximal increase of [Ca^2+^]_cyt_ (T_max_) after application of flg22 and Pep-13 in comparison to H_2_O_2_. Values are derived from the data in Figs. 4 and 5A, and 5B. Treatments that resulted in non-significant differences between the two species are indicated (two tailed student’s t-test, *p* ≤ 0.01).

Various studies have demonstrated that Ca^2+^ signals together with an apoplastic ROS-burst play an important role in activating the plant’s pathogen defence system^28,29^. Changes in [Ca^2+^]_cyt_ are thus an early and essential element in intracellular signalling networks after perception of pathogen-associated molecular patterns (PAMPs). Due to the observed species-specific Ca^2+^ transients in response to H_2_O_2_, we decided to also analyse [Ca^2+^]_cyt_ changes in response to two biotic elicitors (Fig. 5a-b). Compared to the abiotic stimuli, responses to the biotic components were generally slower (Fig. 5c) and measurements were taken for longer periods.

### Flg22

Flg22 is a 22 amino acid long fragment of the *Pseudomonas syringae* flagellin whose induction of Ca^2+^ transients and triggering of ROS production has been described in different plant species and tissues^30–32^. While an increase in [Ca^2+^]_cyt_ occurred already at 0.5 µM flg22 in Arabidopsis, a clear peak was observed with concentrations of 1 µM and higher (Fig. 5a, Arabidopsis). A maximal amplitude of less than 0.2 µM [Ca^2+^]_cyt_ was observed at around 160 s after application, similar to what has been described earlier in Arabidopsis^21,33^ and the peak declined slowly (Fig. 5a, Arabidopsis). The [Ca^2+^]_cyt_ transient in potato in response to flg22 looked similar but the response was even slower than in Arabidopsis. Its elevation started only 3 min after application with a peak time around 300s (Fig. 5a, potato and Fig. 5c), which is similar to what was described for barley^21^. In both species, the time it took to reach the maximum amplitude after application of the flg22 stimulus (T_max_) was much longer than the response to the direct H_2_O_2_ application (Fig. 4), independent of the concentration tested (Fig. 5c). This could be due to flg22 inducing ROS production which subsequently, and thus time-delayed, triggers a Ca^2+^ transient.

### Pep-13

We also tested [Ca^2+^]_cyt_ changes in response to Pep-13, a fragment of a glycoprotein from the late blight-causing *Phytophthora infestans*. Arabidopsis showed only a very minor increase in [Ca^2+^]_cyt_ of less than 0.04 µM with 5 µM elicitor and no response at higher concentrations (Fig. 5b, Arabidopsis). The response in potato to 5 µM Pep-13 was similar but with a higher amplitude of 0.05 µM. At 20 µM Pep-13, the response was lower and similar to what was observed in Arabidopsis at 5 µM (Fig. 5b, potato). The time to the maximum increase in [Ca^2+^]_cyt_ was even longer than with flg-22 and was observed 6 min after application (Fig. 5c). What we did not observe was a slower response of potato compared to Arabidopsis as seen for flg22. (Fig. 5c)

### In vivo imaging of H2O_2_induced redox changes using potato biosensor plants

**Fig. 6:**
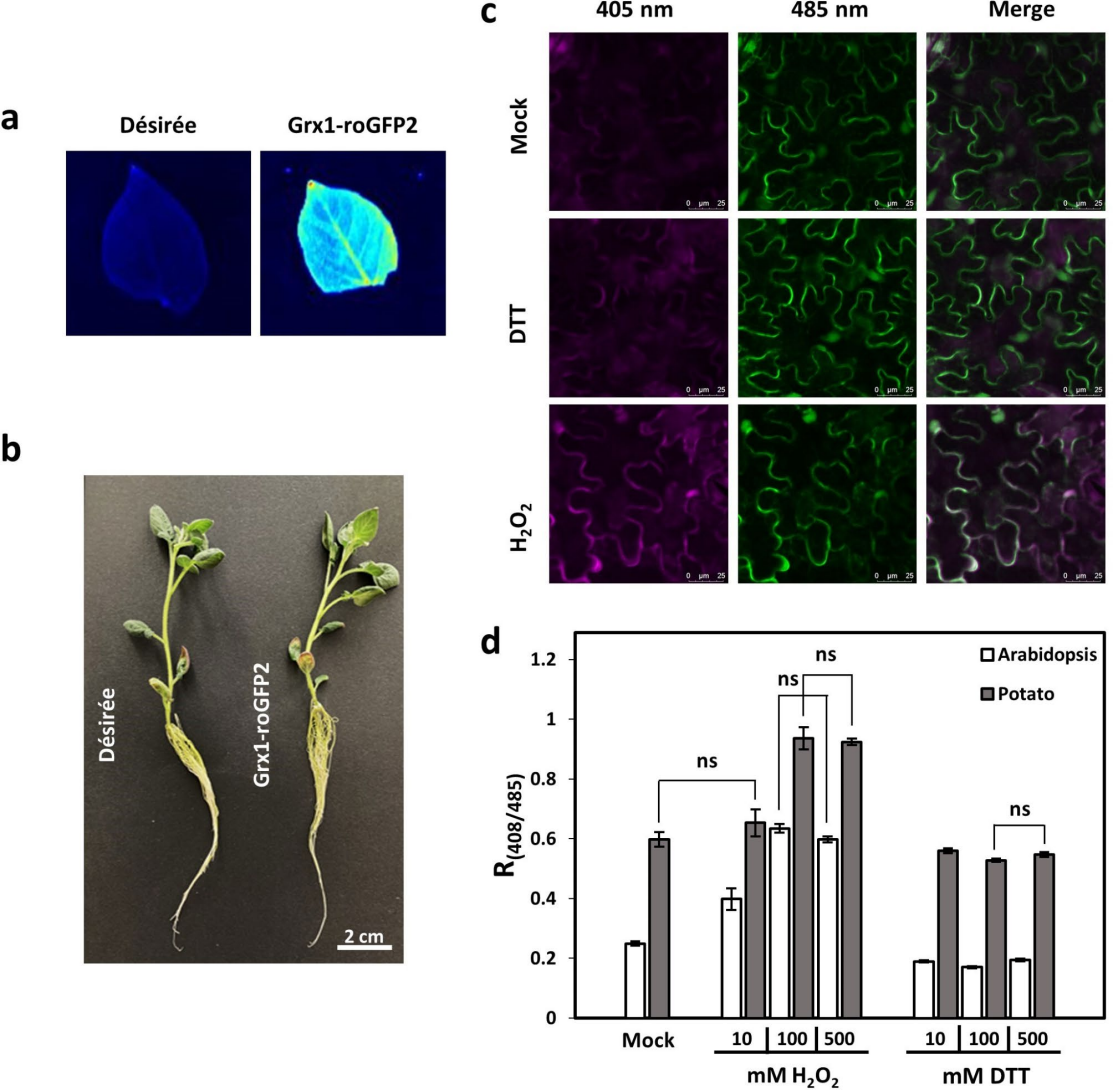
Cytosolic redox status of soil-grown potato and Arabidopsis plants. (**a**) Image of the abaxial side of full mature leaves of wild type and St-Grx1-roGFP2 plants after excitation with blue light (460–485 nm). (**b**) Representative picture of three-week old Désirée (wild type) and St-Grx1-roGFP2 potato plants. (**c**) Confocal images of St-Grx1-roGFP2 plants after sequential excitation at 405 nm, 485 nm, and the merge of both channels. Leaf discs were incubated for 10 min in either mock solution, 100 mM DTT or 100 mM H_2_O_2_. (**d**) Ratiometric measurement of emission at 535 ± 15 nm after sequential excitation at 405 nm and 485 nm. Leaf discs were placed with abaxial side upwards into 96-well plates and emission was measured 10 min after application of imaging buffer (mock), H_2_O_2_ (10, 100 or 500 mM) or DTT (10, 100 or 500 mM) (*n* = 9, mean ± SE). Treatments that resulted in non-significant differences within the same species are indicated (two tailed student’s t-test, *p* ≤ 0.01).

When analysing the Ca^2+^ response of Arabidopsis and potato to H_2_O_2,_ we observed a marked difference in timing and amplitude between the two species (Fig. 4). We were wondering whether this is caused by *bona fide* differences in the molecular response to equivalent redox changes, or whether our treatments induced different redox changes in the two species. To address this question, we expressed the redox-sensitive Grx1-roGFP2 sensor under control of the 35S CaMV promoter in the same cultivar (Désirée) as used for the Ca^2+^ analyses (Fig. 6; Supplementary Fig. S2-S4). Several independent lines were obtained that showed a strong and reliable emission at 510 nm upon excitation at 485 nm under reducing conditions (Supplementary Fig. S2). Grx1-roGFP2 line #29 was used for subsequent analysis. It revealed a clear fluorescence signal throughout the leaf with stronger signals in the main leaf veins when imaged under blue light (460–490 nm) using a fluorescence imager (Fig. 6a). Expression of Grx1-roGFP2 had no visible effect on the growth of this line compared to wild type and Western Blot analysis confirmed the presence of the Grx1-roGFP2 protein (Fig. 6b; Supplementary Fig. S3).

Confocal laser scanning microscopy of leaf discs of Grx1-roGFP2 confirmed the cytosolic localisation of the sensor (Fig. 6c). Reducing conditions induced by 100 mM DTT only resulted in minor changes of the fluorescence signals observed at 535 ± 15 nm after excitation at either 405–485 nm. Oxidation by 100 mM H_2_O_2_ lowered the emission upon excitation at 485 nm and clearly increased the emission upon excitation at 405 nm (Fig. 6c). Together this shows that the sensor line can be used to assess the cytosolic oxidation status.

Further analyses were performed using the same plate luminometer employed for the aequorin measurements. Comparison of soil grown Grx1-roGFP2 plants from potato with Grx1-roGFP2-expressing Arabidopsis^34^ without any stress treatment showed a nearly three times higher ratio of 405/485 nm fluorescence in potato (Fig. 6d, mock). The biosensor is most sensitive for the oxidation status of the small antioxidant glutathione (GSH); thus, the data indicate a higher basal GSH oxidation in potato. Treatment of Arabidopsis and potato with DTT revealed only minor changes in the 405/485 nm ratio with 10 mM DTT and no further decrease with 100 mM DTT or higher (Fig. 6d; Supplementary Fig. S4). This confirms the data from the microscopic analysis of potato (Fig. 6c) and demonstrates that 100 mM DTT fully reduces the leaf cytosolic GSH for both species.

Arabidopsis and potato show slightly differences under treatment with H_2_O_2_. Both species reached a maximum 400/485 nm ratio and thus full oxidation of the cytosol at 100 mM H_2_O_2_. However, upon treatment with 10 mM H_2_O_2_, Arabidopsis leaf cells showed a significant increase in ratio of 405/485 nm fluorescence, while in potato no significant difference to the mock value was observed (Fig. 6d). After treatment with 100 mM H_2_O_2_, the ratio of 400/485 nm fluorescence showed a further increase in Arabidopsis and a significant increase in potato. No further increase in ratio was observed with higher H_2_O_2_ concentrations for either species. Thus, the full dynamic range could be achieved for both species with 10 mM DTT and 100 mM H_2_O_2_, however, while in Arabidopsis the ratio of 405/485 nm fluorescence increased from 0.2 to 0.6 (dynamic range of ~ 3), potato only showed less than a duplicating of the ratio from 0.55 to 0.9 (Fig. 6d). This indicates quite a strong difference in GSH oxidation between Arabidopsis and potato and the high level of GSH oxidation might play a part in the lower H_2_O_2_-induced Ca^2+^ response that we observed in potato (Fig. 4b).

## Discussion

Transient changes in the concentration of [Ca^2+^]_cyt_ are among the earliest responses of plant cells to many environmental stresses. Intriguingly, the exact dynamics of the Ca^2+^ signature not only differs depending on the stimulus but can also differ between stress acclimated and non-acclimated plants^9^. This raises the exciting possibility that screening of specific Ca^2+^ signatures could identify promising varieties for improved crop breeding. However, before such an approach could be incorporated into breeding programmes, baseline Ca^2+^ responses for the desired crop need to be investigated. Considering the importance of Ca^2+^ signalling as a very early component of plant stress responses, surprisingly few investigations using GECIs have so far been performed with plants other than Arabidopsis^18–21,35^. As part of an large-scale analysis of molecular and phenotypical responses of potatoes to environmental stress (https://adapt.univie.ac.at/), we thus investigated stress-dependent changes in [Ca^2+^]_cyt_ in the moderate stress-resistant potato variety Désirée. To better put our data in the context of what is known in the model plant Arabidopsis, we measured all stress responses in parallel between both plants. This avoids as much as possible issues, such as difference in plant age, growth conditions etc. when comparing to already published data. Although the apoaequorin expression level was lower and aequorin-derived photon counts in potato reached only about 25% as those observed in Arabidopsis (supplementary Fig. S1a and b), we could measure well-defined responses in the potato line #20 with stress-induced photon counts well above baseline levels. Thus, we believe that the differences we observed represent species-specific responses of potato and Arabidopsis.

With regards to NaCl, the differences between Arabidopsis and potato mostly pertained to the shape of the response curve at different concentrations (Fig. 2). While both organisms showed a clear dose dependent response to NaCl, Arabidopsis exhibited a strong shift in the maximum peak which was reached earlier with higher concentrations. By contrast, this effect of concentration was very minor in potato. Ultimately, both species reached a similar shape of the response curve, however, potato required about double the NaCl concentration for a maximal response and even then, a much lower peak value for [Ca^2+^]_cyt_ was recorded. The much lower NaCl response in potato is remarkable since potato plants have been described as rather sensitive to salt stress with a soil salinity threshold of 1.7 dSm^− 1^. This is remarkably low in comparison to more NaCl-insensitive crops such as wheat (6 dSm^−1^) and barley (8 dSm^− 1^)^24^, the latter of which shows a NaCl-induced Ca^2+^ transient in its leaves with a maximum amplitude of around 0.4 µM^21^. The salt overly sensitive (SOS) pathway plays a crucial role in the salt tolerance of plants and involves the activation of a N^+^/H^+^ antiporter by a Ca^2+^-dependent sensor-protein kinase complex^36^. Very little is known about the SOS pathway in potato^37^. Whether the lower Ca^2+^ transient in potato leads to less activation of the SOS pathway and therefore impacts subsequent salt tolerance remains to be tested, especially because Arabidopsis displays a high NaCl-induced Ca^2+^ transient but is also considered as salt sensitive. While such species-specific sensitivity to salt might have an impact on the Ca^2+^ responses they are not sufficient to fully explain these observations, especially in light of the fact that salt stress occurs primarily as soil salinity and is sensed in the roots^38^. Consequently, very little is known about salt sensing in leaves and shoots. Another reason for the observed differences in Ca^2+^ responses could lie in different physical surface properties of the leaves that affect the uptake of NaCl, however NaCl penetration into the tissue cannot be easily accessed experimentally.

In contrast to NaCl, mannitol is a non-ionic, osmotic substance. It is used to mimic drought stress since the latter is often not applicable in experimental set-ups, such as the one used in this study. Increase in [Ca^2+^]_cyt_ in response to mannitol has been shown in several previous studies for whole young seedlings of Arabidopsis^13,39,40^. However, when roots and shoots were analysed separately, it was shown that the response occurred exclusively in roots while no [Ca^2+^]_cyt_ transient could be observed in leaves neither in Arabidopsis nor in barley^21,41^. In this study the lack of response in leaves could be confirmed for Arabidopsis also in case of soil-grown plants (Fig. 3). By contrast, a broad [Ca^2+^]_cyt_ transient could be observed in response to 400 mM in the leaf tissue of potato. The underlaying mechanisms for these species-dependent responses to mannitol need to be further investigated at a molecular level.

With regards to H_2_O_2_, it was most remarkable that for both species rather high concentrations were required to elicit a response. In experiments using leaves from young seedlings grown on sterile medium, 10–15 mM H_2_O_2_ was sufficient for a maximal response in Arabidopsis and barley^21^, while in the leaves of soil-grown, older plants used in this study the amplitude of [Ca^2+^]_cyt_ increased up to 100 mM H_2_O_2_ for both Arabidopsis and potato. This might be due to a difference in penetration of H_2_O_2_ into the tissue of the older, soil-grown plants. Moreover, while Arabidopsis showed response curves with a dose-dependent increase in the maximal amplitude but identical shape, the response curve in potato became biphasic with two overlapping broad peaks of similar amplitude. The timing of the first of these two peaks matched the single peak seen in Arabidopsis indicating that they are caused by the same initial response to H_2_O_2_. The second peak in potato could be attributed to secondary responses that are activated by the initial increase in [Ca^2+^]_cyt_. Biphasic signature have been described before also for Arabidopsis in response to ozone or even H_2_O_2_^14,42^ however, in these studies, whole seedlings including roots were used and the second peak occurs much later (~ 600s after application) than what we observe with potato. Thus, the molecular basis for the biphasic response of potato is likely different. In this regard, the differences we measured for the basal oxidative state of the cytosol using the GRX1-roGFP2 sensor lines for Arabidopsis and potato (Fig. 6d) is interesting. Our data shows that both species have the potential to reach a similar increase of the oxidative state (+ 0.4 R_405/485_) after being stimulated with 100 mM H_2_O_2_. However, the reason behind the higher basal level of R_405/485_ (0.2 vs. 0.55) in potato remains unclear. Former research with a chloroplast-targeted GRX1-roGFP2 probe in potato^43^ revealed that the basal oxidative state of the chloroplast was higher for older leaves compared to younger leaves and they speculated that this may be due to an increase in photosynthesis efficiency and decrease in photoprotection.

Differences between Arabidopsis and potato where also observed for the two PAMPs, flg22 and Pep-13. Most remarkable was the response to Pep-13, which contrary to most other stimuli we tested in the present study, elicited a higher [Ca^2+^]_cyt_ increase in potato than in Arabidopsis, suggesting that potato has a higher sensitivity to Pep-13 and thus *Phytophthora infestans*. Indeed, late blight, caused by *Phytophthora infestans*, is the most devastating disease of global potato production^44^. It was however shown before that Pep-13 elicits a defence response against late blight in parsley including a [Ca^2+^]_cyt_ transient with a peak of about 0.8 µM at around 150 s^45,46^. This response is faster and stronger than what we observed in Arabidopsis and potato (Fig. 5b). This might be the result of the different types of sample tissue used (parsley suspension cells vs. leaf tissue from soil grown plants) or could indicate a high susceptibility of parsley to *Phytophthora*. Indeed, severe yield loss of parsley due to root rot caused by *Phytophthora cryptogea* has been described^47^.

Overall, when comparing the interplay between species- and stimulus-dependent responses, no simple conclusion as to the driving factor of the observed differences can be drawn. While issues such as difference in stimuli-penetration need to be addressed methodically, it can be expected that species-specific differences in Ca^2+^ transients affect the downstream mechanism of perception and translation of the stimulus into cellular responses. Mechanistically, Ca^2+^ signatures are shaped by the activity of Ca^2+^ transporters and channels, as well as Ca^2+^-buffering systems^48^.

The phylogenetic relationship between Ca^2+^ transport proteins across a range of plant species has been determined^49^ and based on this analysis, we identified similar sequences in the potato reference genome Phureja DM1-3 v6.1 (Supplementary Table S1). Notably, the potato genome seems to encode more GLRs (27 in potato vs. 20 in Arabidopsis), MCA channels (7 vs. 2) and Ca^2+^-ATPases (21 vs. 14). However, given the overall higher number of annotated protein-coding genes in potato compared to the Arabidopsis genome^50^ these differences are not significant (chi-square test, *p* < 0.05). Nevertheless, variance in the Ca^2+^ toolbox between species might be related to some of the observed difference in stress-induced Ca^2+^ transients and the functionality of the corresponding gene products and the impact of a putatively increased complexity in potato remain to be studied further. With regard to down-stream mechanisms activated by the Ca^2+^ transients, an alteration in amplitude and/or shape could affect how different plants can cope with environmental changes. This could be investigated by analysing the Ca^2+^ response of different varieties of the same species that are more or less susceptible to a certain stress. In that regard, our data can be used to define which stimuli and stimuli concentrations are useful to trigger cytosolic Ca^2+^ changes in the moderate stress-resistant potato variety Désirée and by that offers a basis for future Ca^2+^ related studies in this crop species.

## Methods

### Vector construction and transformation into*S. tuberosum*(cv. Désirée)

To generate potato lines of the cultivar Désirée with constitutive expression of *apoaequorin* (St-AEQ_cyt_), the pMAQ2 vector carrying the coding region of *apoaequorin* down-stream of the cauliflower mosaic virus (CaMV) 35S promoter was used^12^. For the generation of Désirée lines with constitutive expression of redox-sensitive *roGFP2* fused with human *glutaredoxin 1* (St-Grx1-roGPF2) a previous described fusion construct^51^ was inserted into pBINar upstream of the CaMV 35S promoter. Both constructs were introduced into the potato cultivar Désirée as described previously^52^.

### Plant material and growth conditions

In addition to the transgenic potato plants described above, corresponding transgenic *A. thaliana* plants (Col-0) expressing either cytosolic *apoaequorin* (At-AEQ_cyt_;^12^) or *Grx1*-*roGFP2* (St-Grx1-roGFP2^53^) under the control of the cauliflower mosaic virus 35S promoter were used.

Potato cuttings/explants were first grown on sterile MS agar (pH 5.7, 2% (w/v) sucrose) for root formation and subsequently transplanted into single pots filled with soil. In case of Arabidopsis, seeds were sown onto the same soil, stratified for 2 days at 4 °C in the dark, and separated after germination into single pots. Potato and Arabidopsis plants were subsequently cultivated side-by-side for 3 weeks in a growth chamber with a temperature of 20 ± 2 °C at a light intensity of ~ 150 µmol photons m^− 2^ s^− 1^ (Philips TLD 18 W of alternating 830/840 light colour) under long day (16 h light/8 h dark) conditions.

### In vitro aequorin reconstitution and quantification

To quantify *apoaequorin* expression in transgenic potato plants, aequorin was reconstituted and chemiluminescence was measured in vitro. Leaf discs (Ø 6 mm) were collected from fully expanded leaves of soil-grown St-AEQ_cyt_ plants and the fresh weight was recorded. The tissue was homogenised in 500 µL extraction buffer (0.5 M NaCl, 9.547 mM beta-mercaptoethanol, 5 mM EDTA, 0.2% (w/v) gelatine, 10 mM Tris-HCl pH 7.4) and cleared by centrifugation in a bench-top centrifuge at 16,300 *g* for 10 min. Supernatants were transferred to new tubes and reconstituted with 1 µM coelenterazine (Biosynth AG, Switzerland) at room temperature in the dark overnight. The following day, 4 µL aliquots were added to 200 µL of 200 mM Tris HCl, 0.5 mM EDTA, pH 7.0 in the wells of a 96 well plate. The relative amount of aequorin in each extract was calculated by measuring photon counts over a 10-second period before and after addition of 200µL of 50 mM CaCl_2_ (final concentration 25 mM), using a plate luminometer (Tristar 3 Multimode Reader, Berthold GmbH). Aequorin abundance was expressed as relative luminescence / fresh weight (arbitrary units/mg).

### Aequorin and GFP immunodetection

Proteins were isolated from leaves of 3-week-old potato plants using Lacus protein isolation buffer (20 mM Tris pH 7.7, 80 mM NaCl, 0.75 mM EDTA, 1 mM CaCl_2_, 5 mM MgCl_2_, 1 mM DTT, 2% (w/v) SDS). The proteins were separated on a 12% SDS-polyacrylamide gel and transferred to nitrocellulose membrane (0.45 μm pore size). After transfer of the proteins the membrane was stained using 0.1% (w/v) Ponceau S in 5% (v/v) glacial acetic acid. Immunodetection was performed using antibodies against aequorin (Abcam, Berlin, Germany) or GFP (Agrisera, AS20 4443) and an ECL detection system with an anti-rabbit secondary antibody coupled to horseradish peroxidase.

### *In planta* reconstitution of apoaequorin and stimuli-induced luminescence measurements

One day before measurements were taken, leaf discs (Ø 6 mm) were collected from ~ 3-week-old plants and floated overnight in the dark at 20 °C in 10 µM coelenterazine (Biosynth AG, Switzerland) for reconstitution. The following day, the leaf discs were transferred individually into a 96-well plate containing 100 µL ddH_2_O. Photon count measurements were performed using a plate luminometer (Tristar 2 Multimode Reader, Berthold GmbH). The basal level of photon counts was measured for 30 s for the abiotic stimuli and 60 s for the PAMPs (Flg22, GenScript Biotech Corporation, The Netherlands; Pep-13, ProteoGenix, France) with an interval of 1 s, followed by application of various stimulants using a stock solution with 2x the final concentration and a volume equal to the starting volume (100 µL), with continuous measuring for a minimum of 240 s after application. Subsequently, the remaining aequorin was discharged by adding 1/3 volume of 3 M CaCl_2_ in 30% (v/v) EtOH resulting in a final concentration of 1 M CaCl_2_ in 10% (v/v) EtOH. Photon counts were then recorded for another 300 s. [Ca^2+^]_cyt_ was calculated based on the photon counts as described previously^54^.

### Measurement of Grx1-roGFP2 fluorescence

To check the expression of Grx1-roGFP2 in leaves of 3-week-old wild type and St-Grx1-roGFP2, plants were imaged via the ChemiDoc MP Imaging System (BioRAD, USA) with a blue light source (460–490 nm) and an exposure time of 0.4 s.

For imaging of Grx1-roGFP2 fluorescence using laser scanning confocal microscopy, leaf discs (Ø 7 mm) of 3-week-old St-Grx1-roGFP2 plants were pre-incubated in imaging buffer (10 mM MES pH 5.8, 10 mM MgCl_2_, 10 mM CaCl_2_, 5 mM KCl) for 30 min and then transferred onto a microscope slide. The samples on the slide were covered with either ddH_2_O, 100 mM H_2_O_2_ or 100 mM DTT and mounted into the light pass of a Leica SP8 lightning (Leica Biosystems, Germany). Images were collected using a 40x lens (HC-PL-APO-C22, Zeiss) in multi-track mode with sequential excitation by 485 nm (1% gain) and 405 nm lasers (5% gain). Emitted roGFP2 fluorescence was detected from 505 to 536 nm 10 min after the treatment started.

roGFP2 fluorescence was further measured in 96 well plates using the Tristar2 LB 942 multimode reader (Berthold, Germany). After cutting the leaf discs (Ø 7 mm) of 3-week-old St-Grx1-roGFP2 and At-Grx1-roGFP2 samples were placed into ddH_2_O for 1 h to rest. For measurements the leaf discs were transferred individually into a 96-well plate containing 100 µL ddH_2_O. Measurements took place with 2-minute intervals for 10 min to measure the basal level of fluorescence, followed by application of different treatments (mock, H_2_O_2_ or DTT) using a stock solution with 2x the final concentration and a volume equal to the starting volume (100 µL), with continuous measuring for a minimum of 70 min after treatment application. Exposure time was set manually to 0.1 s with an alternating excitation at 405 nm (30% lamp energy) and 485 nm (30% lamp energy) using a 535 ± 15 nm emission filter. Redox changes were represented as the ratio of the emission after measurements at both excitation wavelengths (405/485) at 10 min after applying the stimulus.

### Statistics

All Ca^2+^ and redox measurements include data from nine individual replicates. For those nine measurements, the plants were grown independently three times and leaf discs were obtained from three independent plants each time. Pairwise comparisons were performed via students t-test with a significance threshold of *p* ≤ 0.01. All data are represented as means ± SE. Details of the statistical analysis are specified in figure legends.

## Electronic supplementary material

Below is the link to the electronic supplementary material.

Supplementary Material 1

## Data Availability

The data used to support the findings of this study are included within the manuscript or supplementary information files.
