# Supplementary material for: Analysis of abiotic and biotic stress-induced Ca2+ transients in the crop species Solanum tuberosum
Source: Sci Rep. 2024 Nov 11;14:27625. doi: 10.1038/s41598-024-79134-3 (PMC11555376; doi:10.1038/s41598-024-79134-3)
Supplement: Supplementary file 1 — Supplementary Material 1 [file 41598_2024_79134_MOESM1_ESM.pdf]

a

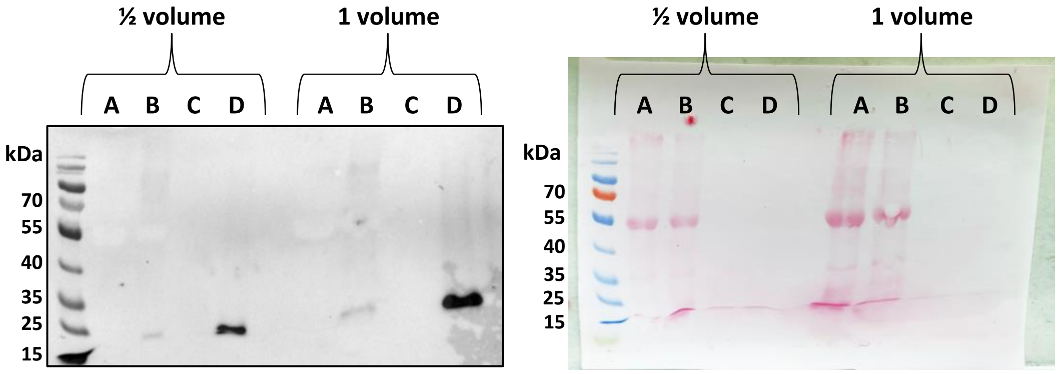

b

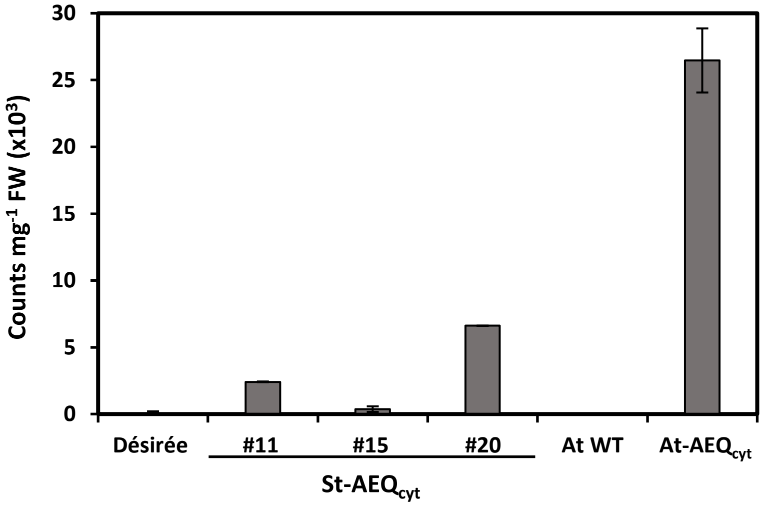

Supplementary Fig. S1: Comparison of abundance of APOAEQUORIN in leaves of At-AEQ$_{cyt}$ and St-AEQ$_{cyt}$#20.

a) The abundance of APOAEQUORIN was determined by immunodetection using a specific antibody against APOAEQUORIN. Wild type plants of potato and Arabidopsis were used as a negative control. A representative blot is shown. The Thermo Scientific™ PageRuler prestained™ protein ladder was used to indicate the size of the detected protein. A: Désirée (wild type), B: St-AEQ$_{cyt}$#20, C: Arabidopsis wild type, D: At-AEQ$_{cyt}$ **b)** Photon counts (average counts 10 seconds after adding 50 mM $CaCl_2$) were measured in leaf extracts from three independent transgenic potato St-AEQ$_{cyt}$ lines and an established AtAEQ$_{cyt}$ line after *in vitro* reconstitution of aequorin with coelenterazine. The counts represent three independent plants and three technical replicates each (n=9, mean ±SE).

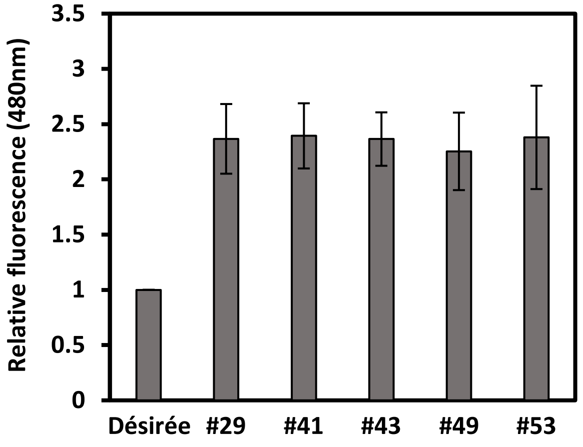

Supplementary Fig. S2: Selection of transgenic 35s::Grx1-roGFP2 potato lines. The redox dependent GFP signal was measured in leaf discs (ø = 7 mm) of Désirée (wild type) and transgenic 35s::Grx1-roGFP2 potato plants upon excitation of 485 nm after treatment with 100 mM DTT (mean ±SE, n = 3)

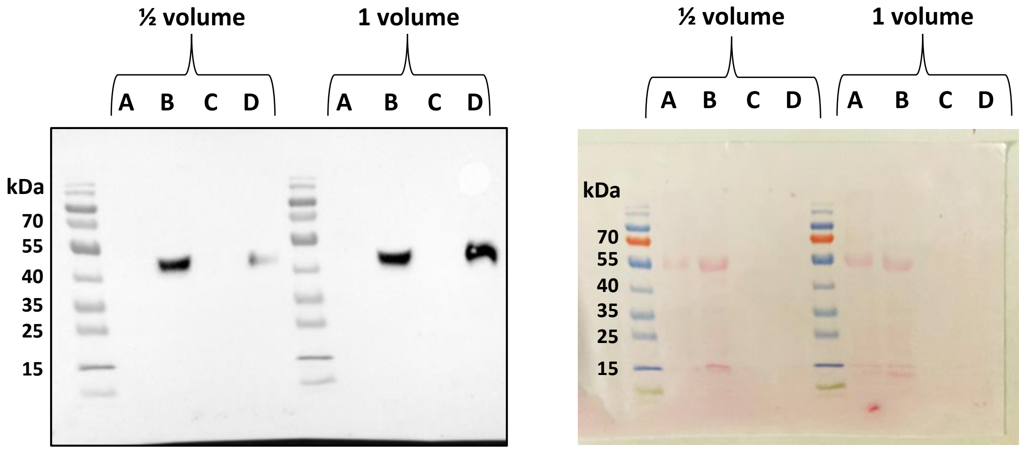

Supplementary Fig. S3: Comparison of abundance of Grx1-roGFP2 in leaves of transgenic Arabidopsis and potato lines. The abundance of Grx1-roGFP2 was determined by immunodetection on leaf extracts using a specific antibody against GFP. Wild type plants of potato and Arabisopsis were used as a negative control. The Thermo Scientific™ PageRuler prestained™ protein ladder was used to indicate the size of the detected protein. A: Désirée (wild type), B: Grx1-roGFP2, C: Arabidopsis (wild type), D: Arabidopsis Grx1-roGFP2

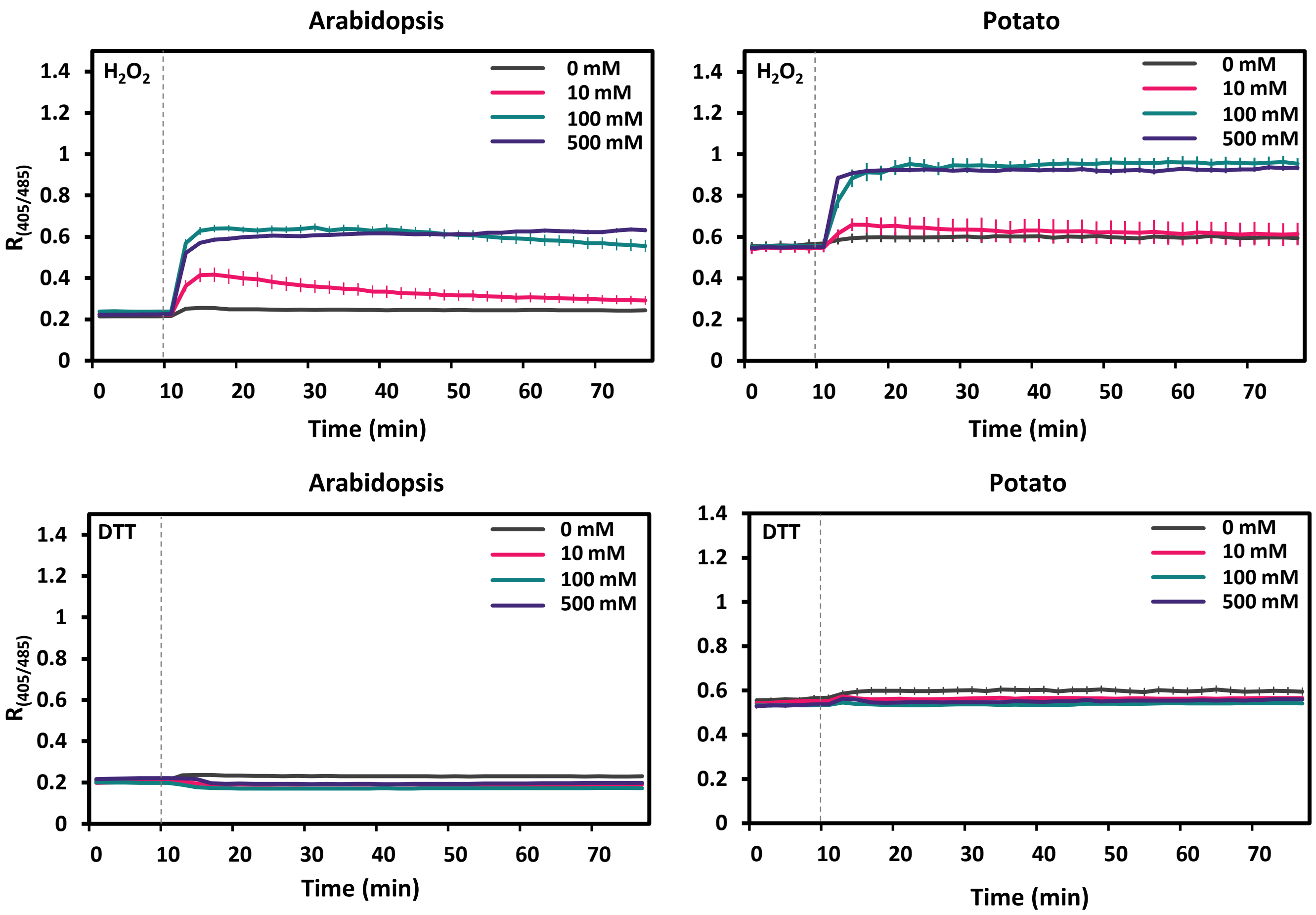

Supplementary figure S4: Time course of changes in $R_{(405/485)}$ upon treatment with different concentrations H_2O_2 and DTT in leaf tissue of Arabidopsis (left) and potato (right) plants. Values are shown as mean ± SE (n = 9). Dashed vertical lines indicate the time point of stimuli injection (10 min).

Supplementary Table S1: Ca^{2+} channels and transporters in Arabidopsis and their orthologues in potato (based on Demidchik et al., 2018).

Family of ion channels or transporters	Number of genes in family (Arabidopsis)	Number of genes in family (potato)
**Ca^{2+} influx systems**		
Cyclic nucleotide-gated channels (CNGCs)	20	23
Ionotropic glutamate receptors (GLRs)	20	27
Two-pore channel (TPC)	1	1
Mechanosensitive-like channels (MSL channels)	10	8
Mid1-complementing activity channels (MCA channels)	2	7
Reduced hyperosmolality-induced [Ca^{2+}] increase 1 channel (OSCA1)	15	14
Piezo channel	1	1
Annexins	8	10
**Ca^{2+} extrusion systems**		
Ca^{2+}-ATPases	14	21
Ca^{2+}/H^+ exchangers	6	6 (+5 CCXs)
